# VELYS robotic-assisted solution achieves more accurate bone cutting and neutral ground mechanical axis compared with an accelerometer-based portable navigation system in ground kinematically aligned total knee arthroplasty

**DOI:** 10.1186/s43019-026-00344-2

**Published:** 2026-08-28

**Authors:** Tomoyuki Matsumoto, Masanori Tsubosaka, Naoki Nakano, Tomoyuki Kamenaga, Yuichi Kuroda, Shinya Hayashi, Ryosuke Kuroda

**Affiliations:** Kobe University Graduate School of Medicine, Kobe, Japan

**Keywords:** Knee, Osteoarthritis, Arthroplasty, VELYS robotic-assisted solution, Kinematic alignment, Ground mechanical axis

## Abstract

**Purpose:**

This study aimed to compare the accuracy of bone cutting, achievement of target alignment, and early clinical outcomes between ground kinematically aligned total knee arthroplasty (KA-TKA) performed using the VELYS^TM^ robotic-assisted solution (VRAS) and that performed using an accelerometer-based portable navigation system (APNS) in patients with varus osteoarthritis.

**Methods:**

This cohort study prospectively enrolled 35 consecutive patients who underwent ground KA-TKA using the VRAS and compared them with 35 consecutive patients treated with the same surgical procedure using the APNS. The primary end point was the accuracy of bone cutting and achievement of target alignment. Clinical outcomes were evaluated at 1 year postoperatively.

**Results:**

The VRAS group demonstrated significantly smaller mean absolute errors from target values, particularly in femoral bone cutting and achievement of a neutral ground mechanical axis, compared with the APNS group (0.7 ± 0.8° versus 1.5 ± 1.1°, 1.6 ± 1.8% versus 3.0 ± 2.4%, *p* < 0.05, respectively). There were no significant differences in range of motion and 2011 Knee Society Score between groups.

**Conclusions:**

Ground KA-TKA performed using the VRAS achieved higher accuracy in bone cutting and more reliable achievement of a neutral ground mechanical axis, compared with the APNS.

## Introduction

Achieving appropriate prosthetic positioning and optimal limb alignment is widely recognized as one of the most important factors influencing clinical outcomes and long-term implant survival following total knee arthroplasty (TKA). In recent years, robotic-assisted systems have emerged as a promising tool to improve the precision of component positioning and limb alignment. Several systematic reviews and metaanalyses have demonstrated that robotic-assisted TKA achieves more accurate component positioning and limb alignment than conventional manual techniques [1, 2]. Among the various robotic systems currently available, the VELYS^TM^ robotic-assisted solution (VRAS; DePuy Synthes) is a relatively new platform and is currently the only image-free system that directly guides the saw handpiece. To date, only a limited number of reports, including preliminary experiences, have evaluated the use of the VRAS in TKA [3, 4].

With growing interest in personalized alignment philosophies, surgeons have sought to reproduce physiological joint lines and native knee kinematics while minimizing soft tissue release. Traditional anatomical alignment (AA)-TKA and several modified procedures derived from unrestricted kinematic alignment (KA)-TKA, such as restricted KA (rKA)-TKA and functional alignment (FA)-TKA, have been proposed. However, recent metaanalyses have failed to demonstrate clear clinical advantages of KA-TKA, AA-TKA, rKA-TKA, or FA-TKA over mechanically aligned TKA [5]. In this context, the hip-to-calcaneus axis, referred to as the ground mechanical axis (MA)[6], which passes through the center of the knee joint in the native knee [7], has gained attention as an alternative alignment reference to the conventional hip-to-talus axis, which is considered the gold standard MA [8, 9]. Based on this concept, Matsumoto et al. modified their surgical strategy for patients with varus osteoarthritis (OA), transitioning from modified KA-TKA [10, 11], where the femoral component is positioned on the cylindrical axis using a calipered technique and the tibial component is consistently placed at 3° of varus, to ground KA-TKA which targets a neutral ground MA [12–14].

Although the benefits of robotic assistance in TKA have been increasingly recognized, its advantages in the context of ground KA-TKA have not yet been reported. Therefore, the purpose of this study was to compare prosthetic orientation and limb alignment, intraoperative soft tissue balance, and early clinical outcomes between ground KA-TKA performed using the VRAS and that performed using an accelerometer-based portable navigation system (APNS) in patients with varus OA. We hypothesized that the VRAS would provide more accurate bone cutting, enabling reliable achievement of a neutral ground MA, and yielding clinical outcomes comparable to those achieved with the APNS in the ground KA-TKA.

## Materials and methods

### Study design and patient characteristics

Inclusion criteria were substantial pain and functional impairment owing to severe knee OA (Kellgren–Lawrence grade 3 or 4) with a functionally intact posterior cruciate ligament (PCL), as assessed by the presence of intercondylar osteophytes on preoperative epicondylar-view radiographs and computed tomography. Exclusion criteria included valgus deformity, severe varus deformity (> 20°), flexion contracture (> 20°), revision TKA, active knee joint infection, and the need for bilateral TKA during the study period. To minimize the influence of postoperative compensatory hindfoot alignment changes [15, 16], patients with a history of ankle or foot surgery, ankle or foot deformity, prior ankle fracture, or inability to stand unassisted on one leg for more than 10 s were also excluded. Severely varus-deformed knees were considered unsuitable for ground KA-TKA owing to the potential for postoperative hindfoot compensation [17].

Between April 2024 and January 2025, 35 consecutive patients meeting these criteria were prospectively enrolled. One patient was excluded after enrollment but prior to surgery owing to coronavirus infection. Consequently, 34 patients underwent ground KA-TKA using the ATTUNE^®^ prosthesis (DePuy Synthes, West Chester, PA, US) with the VELYS^TM^ robotic-assisted solution (VRAS group; DePuy Synthes, Johnson & Johnson MedTech, Warsaw, NJ, USA). A consecutive cohort of 35 patients who met identical inclusion and exclusion criteria and underwent ground KA-TKA using the same prosthesis with an accelerometer-based portable navigation system (APNS group; KneeAlign 2, OrthAlign Inc., Aliso Viejo, CA, USA) between May 2020 and June 2023 served as the control group.

All procedures were performed by a single senior surgeon with more than 15 years of experience in TKA. Baseline demographic and clinical characteristics, including age, sex, body mass index, preoperative deformity, knee phenotype based on the coronal plane alignment of the knee (CPAK) classification [18], lateral distal femoral angle (LDFA), medial proximal tibial angle (MPTA), range of motion (ROM), and 2011 Knee Society Score (KSS), showed no significant differences between groups (Table 1).

**Table 1:** Patient preoperative demographic data

	VRAS (*n* = 34)	APNS (*n* = 35)	*p*-value
Age (years)	76.9 ± 7.5	76.0 ± 8.4	0.662
Female sex, *n* (%)	32 (94.1)	27 (77.1)	0.084
Body mass index (kg/m^2^)	25.8 ± 3.5	27.5 ± 9.8	0.369
Varus deformity (°) #	9.8 ± 4.4	10.8 ± 3.8	0.347
CPAK classification, *n* (%)			
Type I	28 (82.4)	27 (77.1)	0.766
Type II	3 (8.8)	8 (22.9)	0.188
Type IV	1 (2.9)	0 (0.0)	0.493
LDFA (°)	88.6 ± 1.4	88.4 ± 1.3	0.463
MPTA (°)	83.5 ± 2.0	83.5 ± 2.0	0.906
Range of motion (°)			
Extension	−10.3 ± 5.3	−11.7 ± 3.9	0.220
Flexion	118.6 ± 11.4	121.7 ± 13.1	0.300
2011 KSS			
Objective indicator	66.8 ± 5.7	67.7 ± 9.3	0.644
Patient satisfaction	14.7 ± 5.5	15.8 ± 6.5	0.450
Functional activity score	46.6 ± 18.9	52.6 ± 16.5	0.174

Data are presented as mean ± standard deviation. #Positive values indicate varus alignment

*VRAS* VELYS robotic-assisted solution, *APN* accelerometer-based portable navigation system, *CPAK* coronal plane alignment of the knee, *LDFA* lateral distal femoral angle, *MPTA* medial proximal tibial angle, *KSS* Knee Society Score

### Operative procedures

**Fig. 1:**
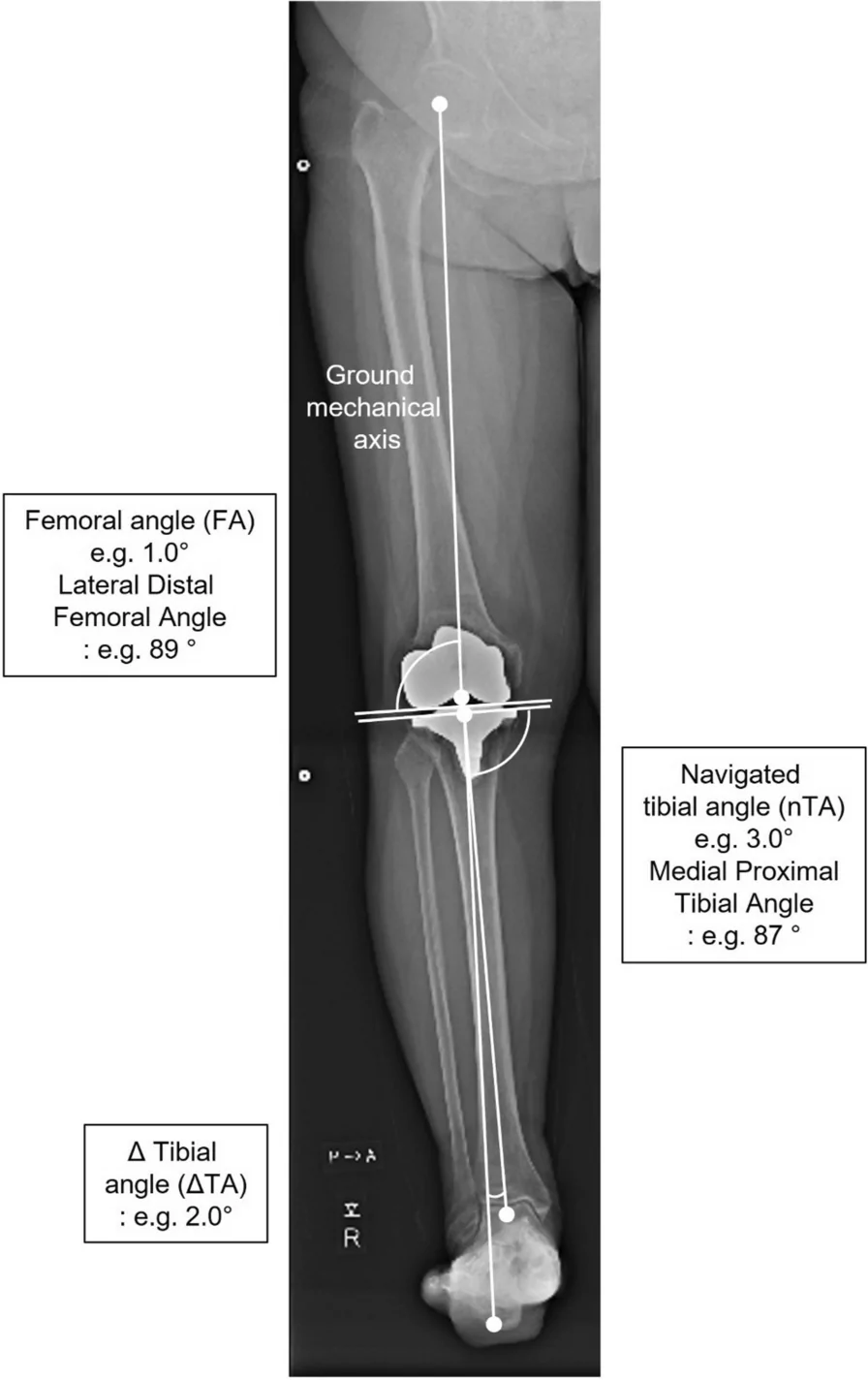
Assessment of the ground mechanical axis. The mechanical axis drawn from the hip center to the center of the distal femoral cut line generally results in valgus alignment relative to the perpendicular line of the distal femoral cut. In this representative case, the femoral angle (FA) was 1.0° valgus. The proximal tibial cut line was simulated to neutralize the FA relative to the ground mechanical axis defined from the center of the proximal tibial cut line to the lowest point of the calcaneus. Both the robotic-assisted system and the navigation system reference the ankle center rather than the lowest point of the calcaneus. Consequently, the mechanical axis referenced by these systems is typically medial to the ground mechanical axis. If the ankle center is located 2.0° medial to the calcaneus (*Δ*TA = 2.0° varus) and the FA is 1.0° valgus (TA = 1.0° varus), the navigated tibial cut angle should be set at 3.0° varus (*n*TA = 3.0°)

Preoperative simulation of ground KA-TKA was performed using full-length standing coronal radiographs that included the calcaneus [12–14]. The distal femoral and proximal tibial cut lines were determined during this simulation. The distal femoral cut was planned using the calipered technique. The femoral angle (FA) was defined as the angle between the femoral mechanical axis (MA) and a line perpendicular to the distal femoral cut, which typically corresponds to the valgus angle in varus-type OA. For tibial planning, the proximal tibial cut was set to neutralize the FA relative to the ground MA of the tibia, defined as the line connecting the center of the proximal tibial cut to the lowest point of the calcaneus. If the FA was 1° valgus relative to the femoral MA, a 1° varus tibial cut relative to the ground MA of the tibia—defined as the tibial angle (TA)—was planned. Because both the VRAS and APNS reference the ankle center rather than the calcaneal contact point, ΔTA was defined as the angular difference between the tibial MA and the ground MA. In general, the calcaneus is located lateral to the ankle center. For example, if FA was 1° valgus (TA = 1° varus) and the calcaneus was positioned 2.0° lateral to the ankle center (*Δ*TA = 2.0° varus), the navigated tibial angle (*n*TA) was set to 3.0° varus. Postoperative alignment parameters corresponding to this example are shown in Fig. 1. The relationship was calculated as follows:

*n*TA (varus) = TA (varus, derived from FA [valgus]) + *Δ*TA (varus).

After tourniquet inflation to 250 mmHg, a medial parapatellar arthrotomy was performed. All TKAs were conducted using an extension-gap-first technique. Following intraoperative confirmation of a functional PCL, the PCL insertion was preserved and cruciate-retaining (CR) TKA was performed.

In ground KA-TKA, the distal femoral cut was first performed using either the VRAS or APNS in accordance with the manufacturers’ instructions and the calipered technique [19]. Minimal medial soft tissue release was performed to maintain medial stability. Femoral osteotomies were completed after compensating for cartilage wear from the distal and posterior femoral condyles with a resection thickness equal to the femoral component thickness (9 mm). Femoral rotation relative to the posterior condylar axis was set to 0°. Based on the FA value confirmed by preoperative planning and intraoperative feedback from the VRAS or APNS, the *n*TA was determined to target a neutral ground MA while preserving the original posterior tibial slope (up to 10°), as planned preoperatively. Thus, both distal femoral and proximal tibial resections were performed with reference to preoperative simulation and intraoperative measurements.

### Radiographic measurements

As the primary end point, prosthetic orientation, limb alignment, and the accuracy of bone cutting and alignment achievement were compared between groups. Full-length standing coronal radiographs including the calcaneus were obtained preoperatively and at 1 month postoperatively to evaluate the ground MA, as previously described [10, 20]. Radiographs were acquired with patients standing on one leg on a radiolucent platform while facing a long-film cassette. To visualize the lowest point of the calcaneus, the cassette was positioned such that its lower edge extended beyond the edge of the platform. The hip–knee–ankle (HKA) angle, LDFA, MPTA, and joint line orientation angle (JLOA) relative to the ground during single-leg standing were measured and compared between groups. The ground MA ratio of the knee joint, defined by the intersection of the ground MA (line from the hip center to the lowest point of the calcaneus) with the tibial plateau, was also evaluated. To assess measurement reliability, two investigators independently performed radiographic measurements twice on 20 randomly selected radiographs. Intraobserver and interobserver reliabilities were evaluated using intraclass correlation coefficients (ICCs), which exceeded 0.85 for all parameters (range, 0.86–0.95). Based on this high reliability, measurements from a single investigator were used for statistical analyses.

Bone-cutting accuracy was assessed by comparing mean absolute errors from the surgical plan for LDFA, MPTA, and ground MA ratio between groups. In addition, the numbers of outliers exceeding ± 2° from target values for LDFA and MPTA and ± 5% from the neutral ground MA (50%) were compared.

### Intraoperative measurement of soft tissue balance

Intraoperative soft tissue balance was assessed using an offset-type tensor that allows evaluation throughout the ROM with femoral component placement and patellofemoral joint reduction [21, 22]. The tensor consists of an upper seesaw plate, a lower platform plate with a spike, and an extraarticular main body. After bone resection and soft tissue release, the tensor was fixed to the proximal tibia. This device enables measurement of the joint component gap and varus/valgus ligament balance while applying a constant distraction force, with minimum measurement scales of 1 mm and 1°, respectively. The tensor allows intraoperative reproduction of postoperative patellofemoral and tibiofemoral alignment, and its reliability has been previously validated with high intraobserver and interobserver correlation coefficients [23].

The intraoperative soft tissue balancing strategy aimed to achieve medial stability with a relatively loose flexion gap and moderate lateral laxity to optimize clinical outcomes [24]. Stepwise medial soft tissue release of the posteromedial capsule and deep layer of the medial collateral ligament was performed as needed, while residual lateral laxity was permitted. A relatively loose flexion gap is reported to be important for the acquisition of postoperative high flexion angle in CR TKA [25]. If the flexion gap was tighter than the extension gap, partial PCL release from the tibial side was performed. Before final implantation, the tensor was fixed, and the femoral trial component and patellofemoral joint were reduced by temporarily suturing the medial parapatellar arthrotomy. Soft tissue balance parameters, including joint component gap (mm) and varus/valgus ligament balance (°), were measured at 0°, 10°, 30°, 60°, 90°, and 120° of knee flexion under a distraction force of 40 lbs. Measurements were repeated until consistent values were obtained to minimize errors related to creep elongation of surrounding soft tissues. A distraction force of 40 lbs was selected on the basis of preliminary clinical studies demonstrating reproduction of the full-extension joint gap corresponding to the insert thickness.

### Clinical evaluations

Clinical evaluations were performed at 1 year postoperatively. In the VRAS group, one patient withdrew 3 months postoperatively owing to colorectal and prostate cancer treatment, and one patient died 2 months postoperatively from myocardial infarction. The remaining 32 patients were included in the final clinical analysis.

Clinical outcomes included ROM and patient-reported outcomes assessed using the 2011 KSS, which comprises symptoms, patient satisfaction, patient expectations, and functional activities. Objective knee indicator scores—alignment, instability, and joint motion—were evaluated by a surgeon blinded to group allocation. Patient-reported components were completed during outpatient visits. Improvements in ROM and KSS subscale scores (postoperative minus preoperative values) were also analyzed to assess treatment effects. Improvement of patient expectation was not calculated because preoperative and postoperative questionnaires were different.

### Data analyses

All data were normally distributed and are presented as mean ± standard deviation (SD). Statistical analyses were performed using GraphPad Prism (GraphPad Software, San Diego, CA, USA). Unpaired *t*-tests were used to compare radiographic parameters, mean absolute errors, intraoperative soft tissue balance, and clinical outcomes between groups. Differences in the numbers of outliers were analyzed using the *χ*^2^ test. Statistical significance was defined as *p* < 0.05.

A priori power analysis was conducted using G^*^Power 3 (Heinrich Heine University Düsseldorf, Düsseldorf, Germany). Based on a previous study comparing radiological accuracy between robotic-assisted and conventional TKA [26], the effect sizes for HKA angle and femoral component alignment were 0.637 and 0.773, respectively. Accordingly, the estimated sample sizes of 32 (64) and 22 (44) patients were required to achieve a power (1-*β*) of 0.80 with a type I error (*α*) of 0.05.

## Results

### Postoperative radiographic evaluation

**Fig. 2:**
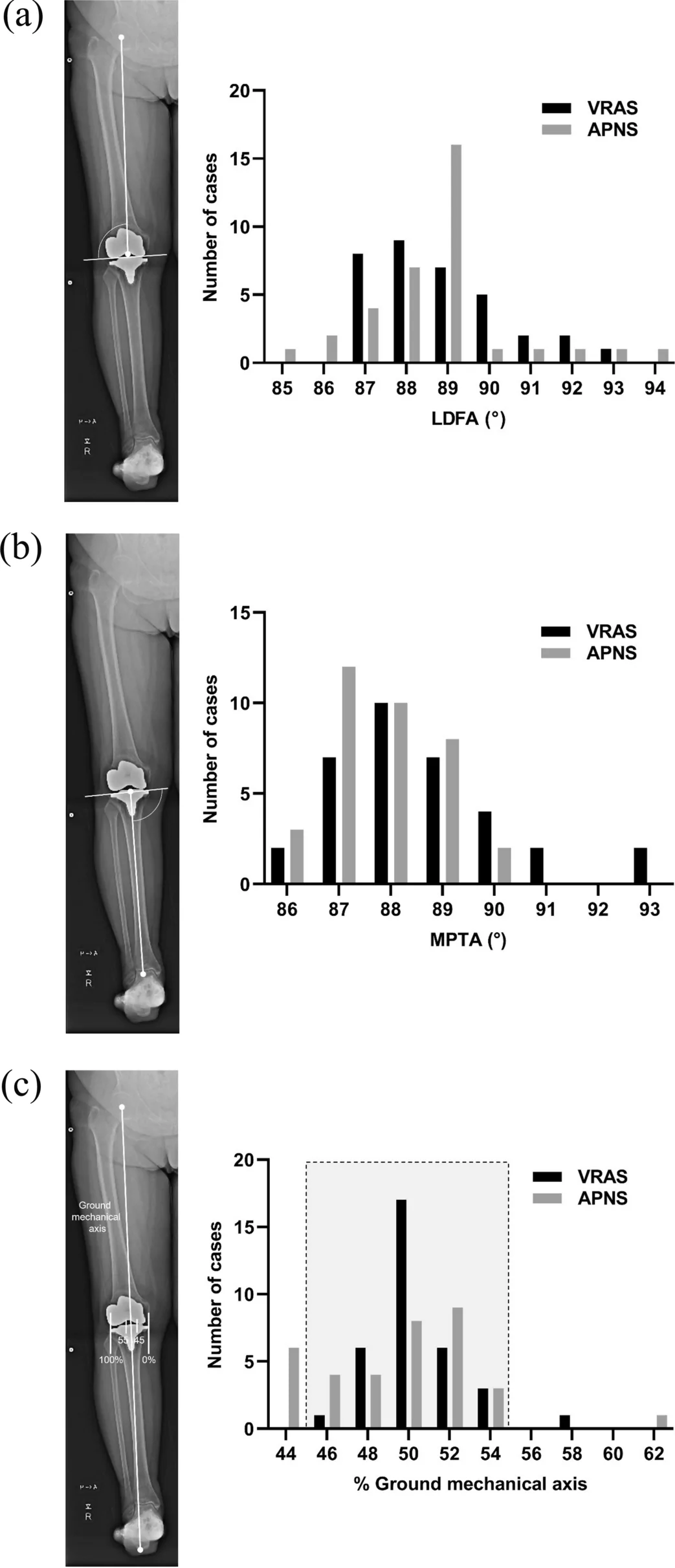
Histograms of radiographic parameters. (A) Lateral distal femoral angle (LDFA). (B) Medial proximal tibial angle (MPTA). (C) Ratio of the ground mechanical axis from the hip center to the lowest point of the calcaneus

Postoperative radiographic parameters are summarized in Table 2. There were no significant differences between groups in HKA angle, LDFA, or JLOA. MPTAs was 88.5 ± 1.6° in the VRAS group and 87.7 ± 1.1° in the APNS group, showing a statistically significant difference (*p* < 0.05). The ground MA ratio was 50.5 ± 2.3% in the VRAS group and 49.5 ± 3.8% in the APNS group, with no significant difference between groups. Histograms of LDFA, MPTA, and ground MA ratio are shown in Fig. 2a–c.

**Table 2:** Postoperative radiographic parameters

	VRAS	APNS	*p*-value
HKA angle (°)	0.4 ± 1.3 varus (3.0 valgus–3.0 varus)	0.9 ± 2.1 varus (3.5 valgus–3.5 varus)	0.192
LDFA (°)	88.7 ± 1.6 (87.0–92.5)	88.7 ± 1.8 (85.0–93.5)	0.934
MPTA (°)	88.5 ± 1.6 (86.0–92.5)	87.7 ± 1.1 (85.5–90.0)	0.025^*^
JLOA (°)	0.0 ± 1.5° varus (3.5 valgus–2.1 varus)	0.7 ± 1.7° varus (2.8 valgus–4.2 varus)	0.072
Ground MA ratio (%)	50.5 ± 2.3 (46.3–58.9)	49.5 ± 3.8 (43.4–62.3)	0.227

Data are presented as mean ± standard deviation (range)

*HKA* hip–knee–ankle, *LDFA* lateral distal femoral angle, *MPTA* medial proximal tibial angle, *JLOA* joint line orientation angle, *MA* mechanical axis

^*^p < 0.05

### Accuracy evaluation

Mean absolute errors from the target values of LDFA, MPTA, and ground MA ratio were 0.7 ± 0.8°, 0.7 ± 0.7°, and 1.6 ± 1.8% in the VRAS group, and 1.5 ± 1.1°, 0.9 ± 0.9°, and 3.0 ± 2.4% in the APNS group, respectively (Table 3). The mean absolute errors for LDFA and ground MA ratio, but not for MPTA, were significantly lower in the VRAS group than in the APNS group (*p* < 0.05).

**Table 3:** Mean absolute errors from surgical plans

	VRAS	APNS	*p*-value
LDFA (°)	0.7 ± 0.8 (0.0–4.0)	1.5 ± 1.1 (0.0–3.0)	0.0004^*^
MPTA (°)	0.7 ± 0.7 (0.0–4.5)	0.9 ± 0.9 (0.0–3.0)	0.209
Ground MA (%)	1.6 ± 1.8 (0.3–8.9)	3.0 ± 2.4 (0.3–12.3)	0.007^*^

Data are presented as mean ± standard deviation (range)

*VRAS* VELYS robotic-assisted solution, *APN* accelerometer-based portable navigation system, *LDFA* lateral distal femoral angle, *MPTA* medial proximal tibial angle, *MA* mechanical axis

^*^p < 0.05

The numbers of outliers exceeding ± 2° from the target values of LDFA and MPTA were 0/34 and 2/34 in the VRAS group and 5/35 and 3/35 in the APNS group, respectively (Fig. 2A, B). Outliers exceeding ± 5% from the neutral ground KA (50%) were observed in 1/34 knees in the VRAS group and 7/35 knees in the APNS group (Fig. 2C). The numbers of outliers for LDFA and ground MA ratio, but not for MPTA, were significantly lower in the VRAS group than the APNS group (*p* = 0.022, 0.027, and 0.667, respectively).

### Intraoperative soft tissue balance

**Fig. 3:**
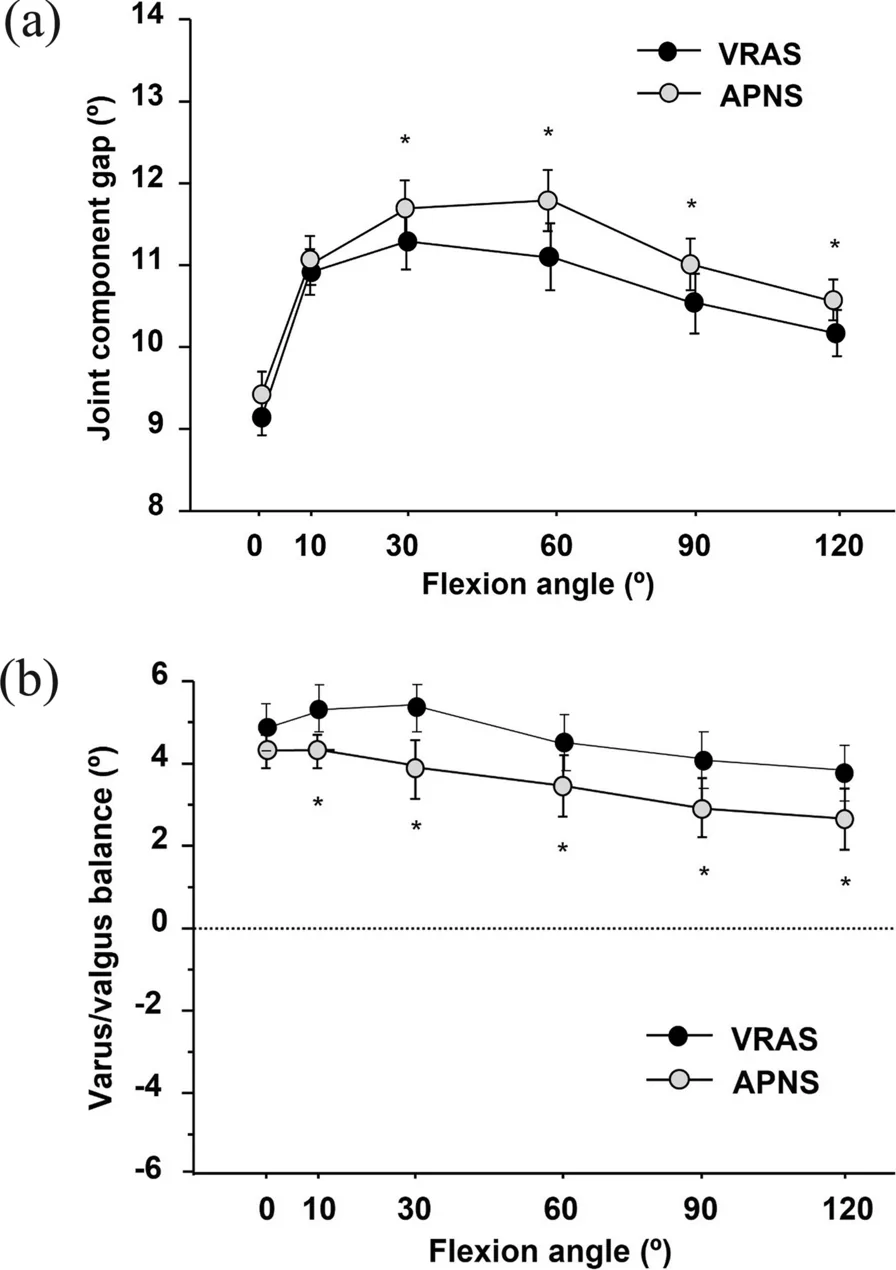
Intraoperative soft tissue balance. (A) Joint component gap. The joint component gaps in the VRAS group were significantly smaller than those in the APNS group during mid-range to deep knee flexion. ^*^p < 0.05. (B) Varus/valgus ligament balance. The VRAS group demonstrated greater lateral laxity throughout the range of motion, except at full extension, compared with the APNS group. ^*^p < 0.05

Mean joint component gaps at 0°, 10°, 30°, 60°, 90°, and 120° of knee flexion were 9.1 ± 0.6, 10.9 ± 0.8, 11.3 ± 1.1, 11.1 ± 1.1, 10.5 ± 1.1, and 10.1 ± 0.8 mm in the VRAS group and 9.4 ± 0.8, 11.1 ± 0.8, 11.7 ± 0.9, 11.8 ± 1.1, 11.0 ± 0.8, and 10.6 ± 0.7 mm in the APNS group, respectively (Table 4). Both groups demonstrated a similar pattern of gap changes throughout the ROM. However, joint component gaps from midflexion to deep flexion (30°–120°) were significantly smaller in the VRAS group than in the APNS group (Fig. 3A).

**Table 4:** Intraoperative soft tissue balance

	VRAS	APNS	*p*-value
Joint component gap (mm)			
0°	9.1 ± 0.6	9.4 ± 0.8	0.114
10°	10.9 ± 0.8	11.1 ± 0.8	0.481
30°	11.3 ± 1.1	11.7 ± 0.9	0.113^*^
60°	11.1 ± 1.1	11.8 ± 1.1	0.013^*^
90°	10.5 ± 1.1	11.0 ± 0.8	0.047^*^
120°	10.1 ± 0.8	10.6 ± 0.7	0.027^*^
Varus/valgus balance (°)			
0°	4.9 ± 1.6	4.3 ± 1.2	0.100
10°	5.3 ± 1.7	4.3 ± 1.2	0.005^*^
30°	5.4 ± 1.6	3.9 ± 2.0	0.002^*^
60°	4.5 ± 2.0	3.4 ± 2.1	0.035^*^
90°	4.1 ± 2.0	2.9 ± 2.1	0.022^*^
120°	3.7 ± 2.0	2.6 ± 2.2	0.033^*^

Data are presented as mean ± standard deviation

*VRAS* VELYS robotic-assisted solution, *APNS* accelerometer-based portable navigation system

^*^p < 0.05

Mean varus/valgus ligament balance at 0°, 10°, 30°, 60°, 90°, and 120° of knee flexion were 4.9 ± 1.6, 5.3 ± 1.7, 5.4 ± 1.6, 4.5 ± 2.0, 4.1 ± 2.0, and 3.7 ± 2.0° in the VRAS group and 4.3 ± 1.2, 4.3 ± 1.2, 3.9 ± 2.0, 3.4 ± 2.1, 2.9 ± 2.1 and 2.6 ± 2.2° in the APNS group, respectively (Table 4). Both groups showed a similar overall pattern across the ROM (positive values indicate varus balance). However, the VRAS group demonstrated significantly greater lateral laxity during flexion (10°–120°) than the APNS group (Fig. 3B).

### Clinical evaluation

The mean postoperative knee extension was −1.1 ± 2.1° (range, −5 to 0°) in the VRAS group and −2.0 ± 4.0° (range, −15 to 0°) in the APNS group. Mean postoperative knee flexion was 125.9 ± 7.7° (range, 95–140°) in the VRAS group and 126.0 ± 10.1° (range, 95–140°) in the APNS group. There were no significant differences between groups in postoperative ROM. While there were no significant differences between groups in objective indicators, patient satisfaction, and patient expectation, the functional activities score was significantly lower in the VRAS group than in the APNS group (73.3 ± 11.4 versus 80.2 ± 13.1, *p* < 0.05). However, improvements in ROM and all subscales of the 2011 KSS including functional activities score did not differ significantly between groups (Table 5).

**Table 5:** Improvements of clinical outcomes

	VRAS	APNS	*p*-value
Range of motion (°)			
Extension	9.4 ± 5.2 (0–15)	9.7 ± 4.2 (−5 to 25)	0.769
Flexion	7.3 ± 10.6 (−35 to 30)	4.3 ± 7.0 (−5 to 15)	0.153
2011 KSS			
Objective knee indicators	28.4 ± 6.1 (18–45)	27.4 ± 7.9 (10–45)	0.585
Patient satisfaction	13.6 ± 7.7 (−2 to 30)	13.0 ± 9.1 (−10 to 34)	0.781
Functional activities	26.7 ± 17.6 (0–67)	27.6 ± 15.9 (−3 to 67)	0.832

Data are presented as mean ± standard deviation

*VRAS* VELYS robotic-assisted solution, *APNS* accelerometer-based portable navigation system, *KSS* Knee Society Score

## Discussion

Given that the VRAS is a relatively new system, only a limited number of studies have investigated its usefulness to date [3, 4]. The present study is the first to investigate the accuracy of the VRAS in bone cutting and its ability to achieve a neutral ground MA in the ground KA-TKA. As hypothesized, compared with the APNS, VRAS provided more accurate bone cutting, particularly on the femoral side, and achieved a significantly higher rate of neutral ground MA, comparable clinical outcomes in the ground KA-TKA.

In the present study, both groups demonstrated similar postoperative radiographic parameters, including HKA, LDFA, MPTA, JLOA, and ground MA ratio. Among them, only MPTA showed a slightly lower value in the VRAS than the APNS group, which may be attributable to differences in target alignment values. However, with respect to bone-cutting accuracy, the VRAS group demonstrated significantly smaller mean absolute errors and significantly fewer ± 2° outliers in LDFA, but not in MPTA, compared with the APNS group. Previous studies comparing tibial bone-cutting accuracy between the APNS and conventional navigation systems (e.g., OrthoPilot) have shown no significant differences in MPTA or posterior slope in modified KA-TKA [27], suggesting sufficient tibial bone-cutting accuracy with the APNS. Consistent with the improved femoral bone-cutting accuracy, the VRAS group also showed significantly smaller mean absolute errors and significantly fewer ± 5% outliers in achieving neutral ground MA. These findings demonstrate that the VRAS enables high accuracy in bone cutting and reliable achievement of neutral ground MA, which was defined as the primary end point of this study. Although the absolute differences were relatively small, they are clinically meaningful in terms of surgical precision and reproducibility. A smaller deviation from the target alignment reduces the risk of outliers, which is crucial for achieving consistent balanced gaps. Furthermore, in the long term, even minor improvements in component positioning may optimize load distribution, thereby potentially reducing polyethylene wear and mitigating the risk of early mechanical failure.

Both groups exhibited a similar pattern of intraoperative soft tissue balance in ground KA-TKA. However, some differences were observed. The VRAS group showed significantly smaller joint component gaps from midflexion to deep flexion and significantly greater lateral laxity throughout the ROM compared with the APNS group. Previous studies have reported that relatively loose, rather than tight, flexion gaps in relation to extension gaps are associated with favorable clinical outcomes, including improved flexion angle [25]. Furthermore, residual lateral laxity, particularly in flexion, has been associated with improved clinical outcomes, including greater knee flexion [24, 28]. Accordingly, the present study suggests that the VRAS successfully achieved balanced soft tissues characterized by relatively loose but not tight flexion gaps and moderate lateral laxity with medial stability, as intended compared with the APNS.

No significant differences were observed between groups in terms of improvements in ROM or subscales of the 2011 KSS. These findings indicate that the improved accuracy of bone cutting and the achievement of neutral ground MA in the VRAS group did not directly translate into superior early-term clinical outcomes. However, the VRAS group demonstrated a tendency toward greater improvement in flexion angle compared with the APNS group. Although this difference did not reach statistical significance, it may be attributable to the relatively loose flexion gaps combined with moderate lateral laxity and preserved medial stability, as discussed above.

Several limitations of this study should be acknowledged. First, this was a prospective single-arm study in comparison with a historical control; therefore, future randomized controlled trials are required. Because of this historical comparison, better outcomes observed in the VRAS group may have been influenced not only by the robotic platform itself but also by increased surgical experience, refinement of perioperative management, and other temporal changes in clinical practice. Second, the study involved a relatively small patient cohort, and patients with severe varus, valgus, or ankle/foot deformities were excluded. The proportion of valgus deformities and severe deformities may have influenced the results and should be investigated further. Third, clinical outcomes were evaluated only up to 1 year postoperatively. As early-term outcomes may not fully reflect long-term clinical relevance, longer follow up is required. Fourth, factors such as cement mantle variability and intraoperative saw toggling may have influenced the radiographic outcomes. Therefore, future studies incorporating both intraoperative validation and postoperative imaging are warranted to isolate the mechanical precision from execution variables. Fifth, while postoperative radiographs remain the standard modality for evaluating gross coronal and sagittal alignment in clinical follow ups, they are inherently limited in capturing intricate, intraoperatively defined three-dimensional kinematic relationships—such as the true dynamic rotational matching or the precise articular-derived tibial posterior slope. Finally, the femoral cut in this study was dependent on the articular surface-reference method which may not accurately reproduce the true cylindrical axis (CA) especially in Asian populations [29, 30]. Consequently, a baseline study investigating how accurately the VRAS algorithm reproduces the true CA remains an essential next step.

In conclusion, the VRAS provided more accurate bone cutting and significantly higher rate of neutral ground MA in the ground KA-TKA compared with the APNS. The use of the VRAS has the potential to facilitate personalized alignment techniques, such as the ground KA-TKA.

## Data Availability

No datasets were generated or analysed during the current study.
